# False-Positive Urine Drug Screening in a Patient on Quetiapine

**DOI:** 10.1192/j.eurpsy.2024.1444

**Published:** 2024-08-27

**Authors:** J. Ying, M. Y. G. Tan

**Affiliations:** East Region, Institute of Mental Health, Singapore, Singapore

## Abstract

**Introduction:**

Urine drug tests are commonly used in psychiatry settings, mainly for the purpose of screening for substance abuse and excluding drug-induced psychiatric disorders. When carefully interpreted, these tests offer critical information for clinical judgement. However, certain psychotropic medications can trigger false-positive results in common urine drug screenings. For example, aripiprazole has been reported to cause false-positive urine amphetamine test results, and haloperidol has been associated with false-positive urine drug tests for lysergic acid diethylamide (LSD). It is clinically significant to recognize some false-positive urine drug results and interpret certain results cautiously in clinical settings.

**Objectives:**

We present a case of false-positive urine drug screening for tricyclic antidepressant (TCA) in a patient on quetiapine and aim to highlight the importance of accurate result interpretation in urine drug tests.

**Methods:**

Details of the case were described. Information was gathered based on medical records.

**Results:**

Mr. A, a 25-year-old construction worker, first presented at our hospital’s emergency room on a Saturday in January 2023. He was brought by the police because he was aggressive and mentioned his colleagues were monitoring him. Being a foreigner, he did not have any prior medical records in our hospital. Urgent blood tests were performed, and organic causes were ruled out. He was started on quetiapine and lorazepam in the emergency room and was then admitted to our hospital.

A urine drug test was ordered on the following Monday, the third day of his admission. Surprisingly his urine drug screening revealed positive results for TCA and benzodiazepines. Initially as the patient was psychotic and could not give reliable history, we considered a few differential diagnoses, such as schizoaffective disorder and major depressive disorder with psychotic features, based on the presumption that TCA had been prescribed by the psychiatrist in Mr. A’s home country. After further treatment, Mr. A became less psychotic and was able to share that he had a past psychiatric history of schizophrenia, but he had stopped antipsychotic medications four months ago.

**Conclusions:**

This case report described a false-positive urine drug test for TCA while the patient was taking quetiapine. In this case, initially other diagnoses, such as schizoaffective disorder, were considered based on the incorrect assumption that patient was taking TCA.

False positive urine drug results can be confusing and misleading for clinicians. This report underscores the possibility of such false positives arising from quetiapine and emphasizes the critical importance of careful result interpretation.

**Disclosure of Interest:**

None Declared

